# Association of *Escherichia coli* O157:H7 Density Change with Hydrogen Peroxide but Not Carbohydrate Concentration in the Leaf Content of Different Lettuce Types and Spinach

**DOI:** 10.3390/foods14040709

**Published:** 2025-02-19

**Authors:** Maria T. Brandl, Sui S. T. Hua, Siov B. L. Sarreal

**Affiliations:** 1 Produce Safety and Microbiology Research Unit, U.S. Department of Agriculture, Agricultural Research Service, Albany, CA 94710, USA; 2 Foodborne Toxin Detection and Protection Research Unit, U.S. Department of Agriculture, Agricultural Research Service, Albany, CA 94710, USA; sylvia.hua@usda.gov (S.S.T.H.); siov.sarreal@usda.gov (S.B.L.S.)

**Keywords:** foodborne pathogen, lettuce, spinach, produce, ROS, oxidative stress, sugars, bioactive compounds, antimicrobial, injury

## Abstract

Leafy greens injuries occur from farm to table, causing leakage of cellular contents that promote the multiplication of foodborne pathogens and impose oxidative stress. Fresh beverages made from blended uncooked fruit and vegetables have become a popular food. The effect of cellular contents of different leafy greens on the multiplication of the important pathogen *Escherichia coli* O157:H7 (EcO157) under temperature abuse was investigated. Leafy greens consisted of spinach and different lettuce types (romaine, iceberg, butterhead, green leaf, and red leaf). Fructose, glucose, and sucrose concentrations in the leaves were quantified by HPLC. H_2_O_2_ concentration was measured via a peroxidase-based assay. Young leaves of iceberg, romaine, and green leaf lettuce held significantly greater total amounts of the three carbohydrates than middle-aged leaves. Except for iceberg and red leaf lettuce, all middle-aged leaves contained greater H_2_O_2_ than young leaves. EcO157 density change in leaf contents over 5 h incubation related neither to individual nor total carbohydrate concentration but was negatively associated with H_2_O_2_ concentration (regression analysis; *p* < 0.05). Given the common use of antioxidants to maintain the organoleptic aspects of homogenized produce beverages and certain fresh-cut produce, the antimicrobial effect of reactive oxygen species may be important to preserve in ensuring their microbial safety.

## 1. Introduction

The wounding of protective barriers provides new opportunities for bacterial growth in most eukaryotic organisms, including plants. Plant tissue injury, during which cellular contents are released, occurs from farm to fork in any plant-derived product. It is particularly intrinsic to harvesting and processing, such as during the production of fresh-cut produce and food preparation. Foodborne pathogens have caused numerous outbreaks associated with fruit and vegetables consumed raw [1,2]. They are reported by the World Health Organization (WHO) and the Food and Agriculture Organization of the United Nations (FAO) to be among the main food vehicles of foodborne Shiga toxin-producing *E. coli* (STEC) illness [3].

We have previously reported that STEC serovar O157:H7 (EcO157), the predominant causal agent of outbreaks linked to lettuce, undergoes more rapid multiplication in the compromised tissue of romaine lettuce leaves than on their intact tissue at a warm temperature [4]. Transcriptomics of EcO157 after a 15 and 30 min incubation in the supernatant of romaine lettuce homogenates indeed showed the rapid upregulation of pathways for the assimilation of plant-derived substrates, particularly those for carbohydrate transport [5]. However, the latter study also revealed the upregulation of genes and regulons involved in adaptation to inhibitory compounds, such as the Mar and OxyR regulons, and other genes with a role in oxidative stress response, multidrug resistance, and antibiotic efflux pumps. While it is well-known that plant tissue actively responds to mechanical damage by the induction of several defense pathways aimed at inhibiting insect herbivory and microbial growth and invasion [6], injured plant tissue also passively leaks cytoplasmic content that may affect microbes positively and negatively.

Plant cells contain a vast array of antimicrobial compounds, many of which also inhibit foodborne pathogens [7,8,9]. Variations in enteric pathogen colonization and survival on different lettuce genotypes have been reported [10,11,12,13]. Plant traits that mediate differential EcO157 colonization of the lettuce phyllosphere in various lettuce cultivars include the cellular reactive oxygen species (ROS) burst [11]. Furthermore, in a screen of lettuce genotypes, differences in peroxidase activity, which may result in the production of H_2_O_2_ and other ROS in plants, were negatively associated with differences in EcO157 decline in cold-stored cut lettuce [10] and in its multiplication in the latex released from cut lettuce stems at harvest [14]. Chemical and physical modulation of wound-generated H_2_O_2_ during cutting of lettuce leaves affected EcO157 survival in the final packaged product [15]. The presence of ROS as an important line of defense against this foodborne pathogen in lettuce tissue and its cellular contents was further corroborated by increased expression of the oxidative stress regulon in EcO157 during a short exposure to cut romaine lettuce and leaf lysates, and by its enhanced survival to treatment with the strong oxidant calcium hypochlorite subsequent to culture in these lysates [5].

In the study, described herein, we sought to assess the effect of the leaf content of various leafy greens on the change in EcO157 density under growth-permissive temperature. We tested the young and middle leaves of five lettuce types representing those most commonly grown in the USA [16,17], as well as bagged baby spinach and bundled spinach, which also contains young and middle leaves. Leaf tissue H_2_O_2_ concentration, an important plant ROS, was measured with the Amplex™ Red peroxidase assay. Treatment of leaf contents with enzymes that degrade H_2_O_2_ was used to corroborate that this ROS was involved in causing the inhibition of EcO157. Additionally, the concentration of growth substrates that are easily assimilated and used by EcO157 was quantified in the leaf contents through HPLC analysis. Given the considerable variation in glucose and fructose content reported among a large collection of lettuce cultivar types [18,19] and the rapid upregulation of the EcO157 sucrose-specific MFS transporter genes, along with those for mono- and polysaccharides in romaine lettuce lysates [5], we specifically focused on these three carbohydrates in our study. The above parameters were then used to determine their relationship to the magnitude of EcO157 density change in the leaf contents over a short period of temperature abuse.

## 2. Materials and Methods

### 2.1. Strains and Culture Conditions Used in This Study

All strains used in this study are listed in Table 1. A spontaneously rifampin-resistant mutant of the EcO157 strain ATCC43888, a non-Shiga toxin-producing strain, was used for the inoculation of lettuce contents (lysates) in this study. For comparative purposes, five EcO157 strains isolated from various environments during the multistate outbreak of infection linked to bagged baby spinach in the USA in 2006 [20] were inoculated as along with strain ATCC43888 into bagged baby spinach lysates. Spontaneous rifampin-resistant mutants of these spinach-linked outbreak strains were isolated in the laboratory and used for selective recovery. All strains were cultured to early log-phase of growth in Luria–Bertani broth—half salt (0.5% NaCl) at 28 °C. Cultures were washed twice in potassium phosphate buffer pH 7.0 (KPB) (10 mM) and resuspended in 1 mM KPB. The suspension was diluted in 1 mM KPB and 20 μL was immediately added to 5 mL of leaf lysates to achieve a concentration of approximately 1 × 10^4^ cells/mL based on OD_600_ of the diluted cell culture.

### 2.2. Leaf Content Preparation and Quantification of EcO157 Change in Density

Lettuce (*Lactuca sativa* L.) types romaine, iceberg, butterhead, and green and red leaf were obtained as mature heads from distributors. Bundled spinach (*Spinacia oleracea* L.) and bagged baby spinach were obtained from retail stores. Lettuce heads and bundled spinach were separated into young and middle leaves and the very outer leaves were discarded. Young and middle leaves corresponded approximately to the 6th (partially expanded) and 12th (fully expanded) positions of the leaves starting from the meristem rosette toward the outer part of the lettuce head, respectively.

Two middle leaves and three young leaves from the same mature lettuce heads or spinach bundles were crushed to a fine paste with a mortar and pestle and as separate composite samples based on the age of the leaves. The resulting lysates were centrifuged immediately at 12,000× *g* at 4 °C for 5 min to pellet cellular debris. The supernatant was filter-sterilized by passing through a 0.45-μm pore-size PVDF (polyvinylidene difluoride) filter (Fisher Scientific, Waltham, MA, USA) and immediately used for inoculation with EcO157 and measurement of H_2_O_2_ and carbohydrate concentrations. This method for lysate production was used successfully in our previously published transcriptome study in which EcO157 was exposed to romaine lettuce cellular contents [5]. For experiments on the remediation of oxidative stress, catalase and superoxide dismutase (Sigma Aldrich, St-Louis, MO, USA) were added to lysates at 150 units/mL and 300 or 3000 units/mL, respectively. All lysates were used immediately for bacterial density measurements and chemical analyses.

The lysates were sampled for EcO157 concentration at 0, 1, 2, 4, and 5 h after inoculation and incubation at 100 rpm at 28 °C to mimic temperature abuse. Plate counts were performed after dilution plating of lysate aliquots onto Luria–Bertani agar containing 100 μg/mL rifampin and plate incubation overnight at 37 °C. Two replicates of composite lysate samples were tested using two different lettuce heads, spinach bundles, or bags of baby spinach. Each lettuce type and spinach were tested in two replicate experiments with plant material obtained from suppliers on separate days. Data from plate counts were used to calculate bacterial density change, here defined as the increase in cell concentration from 0 h at the time of inoculation to 5 h post-incubation.

### 2.3. Carbohydrate Quantification

Carbohydrates in lysates obtained as described above were quantified by high-performance liquid chromatography (HPLC) on an HP ChemStation 1260 Infinity (Agilent Technologies, Santa Clara, CA, USA) equipped with an evaporative light-scattering detector (PL-ELSD 2100). Separation of carbohydrates was achieved with a Prevail^TM^ Carbohydrate ES HPLC column (Alltech Associates, Inc., Deerfield, IL, USA) with acetonitrile: water 75:25 (*v*/*v*) as mobile phase at 1 mL/min flow rate. Peak areas for glucose, fructose, and sucrose in the samples were confirmed by additional injections of samples spiked with HPLC-grade standard compounds. The Chemstation software (LTS, Version 01.11, Agilent Technologies) was used to determine areas under the peaks for lysate samples and glucose, fructose, and sucrose standards, and to calculate the concentrations of these three carbohydrates in the lettuce lysates as mg/mL lysate. The lysate of young and middle leaves was diluted with sterile DDI H_2_O four- and three-fold, respectively, before injection into the HPLC. There were two replicate samples for each lettuce type and leaf age and the experiment was performed twice.

### 2.4. H_2_O_2_ Quantification

Leaves were homogenized and sterile lysates were obtained as described above. H_2_O_2_ quantification was performed with 100 μL lysate immediately after it was obtained using the Amplex™ Red Hydrogen Peroxide/Peroxidase Assay Kit (Thermo Fisher Scientific, Waltham, MA, USA) based on the manufacturer’s instructions. H_2_O_2_ was quantified by fluorescence after 30 min incubation of the assay solution, based on signal intensity and a standard curve generated with different concentrations of freshly prepared H_2_O_2_. A standard curve was generated each time the assay was run. A representative equation for the standard curve was: Y = 7.63 × 10^4^X − 401 (R^2^ = 0.999). Two to five replicate experiments were performed with different lettuce and spinach obtained on different days.

### 2.5. Statistical Analysis

Statistical analysis of the data was performed with Prism version 10.3.0 (GraphPadSoftware, Boston, MA, USA). Every replicate sample in this study was a true biological replicate; therefore, it was obtained from different lettuce heads.

## 3. Results and Discussion

### 3.1. EcO157 Density Change

**Figure 1:**
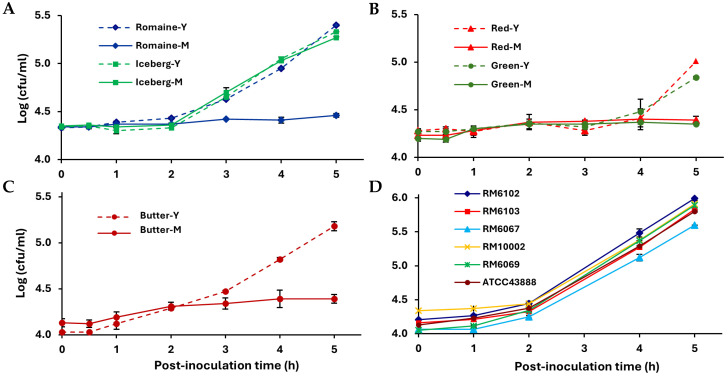
EcO157 population size dynamics in the lysate of young and middle leaves from (**A**–**C**) common lettuce types and (**D**) bagged baby spinach. The lettuce types were romaine, iceberg, red and green leaf lettuce, and butter lettuce. Y and M indicate young and middle leaves, respectively. Lettuce lysates were inoculated with EcO157 ATCC43888. Lysates from bagged baby spinach were inoculated with strain ATCC43888 and five different EcO157 strains isolated from various sources during the 2006 outbreak linked to bagged baby spinach in the USA. Data represent the mean and SEM from two replicate samples, each originating from different lettuce heads or spinach bags. For each replicate, young and middle lettuce leaves were sampled from the same lettuce head.

EcO157 underwent a lag period in all tested leaf lysates (Figure 1) similar to our previous observations in the lysates of middle romaine lettuce leaves [5]. Hence, the change in EcO157 density over 5 h incubation included a lag phase and multiplication phase. The length of the lag phase varied depending on lettuce type and leaf age. Lysates from young leaves caused growth inhibition periods of 0.5 to 4 h before EcO157 multiplication was detected, while minimal growth occurred over the entire 5 h incubation in the middle leaf lysates, except for iceberg lettuce lysates, in which a short lag phase of 2 h was observed irrespective of leaf age (Figure 1). Consequently, young leaf tissue contents promoted an overall significantly greater change in EcO157 density over 5 h than those from middle leaf tissue (Tukey’s multiple comparisons test, *p* < 0.05) (Figure 1A–C and Table 2).

A short lag phase of 0.5 to 2 h was observed also in baby spinach lysates. Strain ATCC43888 achieved a 47-fold increase in cell density in spinach lysates over only 5 h, the greatest increase compared to all lettuce lysates (Tukey’s multiple comparisons test, *p* < 0.05) (Figure 1D and Table 2). Additionally, all EcO157 isolates associated with the spinach-linked outbreak had very similar lag phases and growth rate patterns, irrespective of their source (clinical patients; bags of baby spinach in different states but originating from the same implicated field; and cows or a river near that field) (Table 1) (Figure 1D). The density change in strain ATCC43888 in spinach lysate was also similar to that of the spinach outbreak strains and differed slightly but significantly after 5 h only from that of strains RM6067 and RM6069 (Tukey’s multiple comparisons test; *p* = 0.027 and *p* = 0.007, respectively) (Table 2); both of the latter strains were isolated in the state of Pennsylvania during the 2006 EcO157 outbreak (Table 1). The genetic basis for the difference in behavior between strain ATCC43888 and the latter two outbreak strains may partly lie in differences in the regulation of stress response pathways.

Given that very few cells of this pathogenic *E. coli* may represent a risk to human health [21,22], the approximately 10-fold increase in EcO157 density in lysates from young leaves of most lettuce types and a 47-fold increase in baby spinach lysates over only 5 h suggest the great potential for this pathogen to amplify under temperature abuse to levels of concern in compromised leaf tissue or homogenized leafy greens. Our previous study, in which EcO157 population sizes increased within 4 h at 28 °C by only 2.0-fold on whole romaine lettuce leaves versus 4.5- and 11.1-fold on cut and shredded leaves, respectively, also suggested this greater risk to public health [4]. We observed that after 24 h, EcO157 densities increased to approximately 1 × 10^9^ cells/mL in the lysates of both leaf ages of all lettuce types, and of baby spinach, which amounted to a 10^5^-fold increase. While it is of less relevance to assess the pathogen amplification over a 24 h-long period of temperature abuse at 28 °C, this sampling time in our study nevertheless revealed that the initial conditions causing differential lag phase times among leafy green types and leaf ages do not persist over prolonged incubation. The lag phase, therefore, reflects a period of adaptation to the various oxidative, antimicrobial, and osmotic stresses experienced from exposure to leaf contents, as observed via transcriptomics by Kyle et al. [5]. This growth delay, due to inhibitory conditions, is therefore critical to prevent the amplification of contamination.

### 3.2. Carbohydrates Levels in Lysates

**Figure 2:**
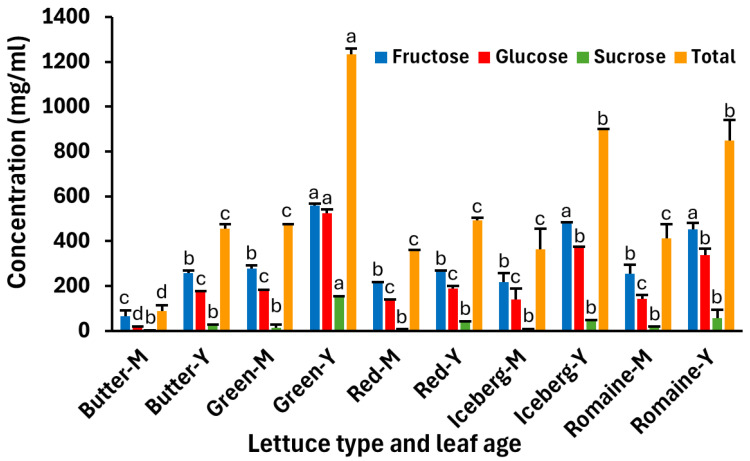
Carbohydrate concentrations in the lysate of young and middle leaves from common lettuce types. The lettuce types were romaine, iceberg, red and green leaf lettuce, and butter lettuce. Y and M indicate young and middle leaves, respectively. The concentrations of fructose, glucose, and sucrose, and the total of the three carbohydrates are illustrated. Data represent the mean and SEM from two replicate samples, each originating from different lettuce heads or spinach bags, and from two replicate experiments. For each replicate, young and middle lettuce leaves were sampled from the same lettuce head. Different letters above bars indicate a significant difference in the means for a given sugar between samples of different leaf ages and lettuce types (Tukey’s multiple comparisons test; *p* < 0.001, 0.05, 0.01, and 0.05, for fructose, glucose, sucrose, and total sugars, respectively).

Glucose and fructose, the product of photosynthesis, are the major carbohydrates present in the leaves of young lettuce plants and in mature heads processed as fresh-cut products, whereas sucrose, lactose, and maltose are present in lower or undetectable amounts [18,19,23]. Similarly, we observed that the concentration of sucrose was much lower than that of glucose and fructose in both young and middle leaves in all types of lettuce investigated herein (Figure 2). Additionally, glucose and fructose concentrations were significantly greater on young than middle leaves, except for red leaf lettuce. However, for sucrose concentration, this leaf age-related pattern was only displayed in green leaf lettuce (Tukey’s multiple comparisons test, *p* < 0.05) (Figure 2). Due to the much greater amount of glucose and fructose than sucrose in the leaves, the trend in total concentration of the three carbohydrates was similar to that of the two monosaccharides (Figure 2 and Table 2).

### 3.3. H_2_O_2_ Levels in Lysates

Although enteric pathogens may benefit from the release of nutrients from damaged cells in injured leaf tissue, they may also encounter the products of secondary metabolism, such as peptides, flavonoids, alkaloids, aliphatic acids, and phenylpropanoids, many of which have antimicrobial activity that also inhibits major foodborne pathogens [7,10,12,24,25]. While the oxidative burst upon plant wounding produces reactive oxygen and nitrogen species, H_2_O_2_ is also continuously generated at various levels in intact plant cells as the byproduct of numerous metabolic pathways [26]. Here, we observed large variations in H_2_O_2_ concentration in the cellular contents of various types and leaf ages of lettuce and spinach, as quantified with the Amplex™ Red assay immediately after the lysates were prepared.

H_2_O_2_ concentrations ranged from 0.43 μM (iceberg—young leaves) to 86.18 μM (red leaf—middle leaves), respectively (Table 2). In a large study of numerous lettuce genotypes, red leaf lettuce had higher anthocyanin content than lettuce types with green leaves [19]. Anthocyanins are known antioxidants that scavenge ROS and protect plants from free radicals generated during abiotic stress, but ROS and anthocyanin levels also modulate each other in apparent cross-talk [27,28]. It is unclear, however, if and how this relationship between anthocyanins and ROS mediated the high levels of H_2_O_2_ observed in red leaf lettuce in our study. Lettuce middle leaves generally had greater H_2_O_2_ concentration than young leaves (Tukey’s multiple comparisons test, *p* < 0.05), with the exception of iceberg middle and young leaves, which contained equally very low amounts (Table 2). In bundled spinach, young leaves also had 2.2-fold lower H_2_O_2_ concentrations than older leaves, and bagged baby spinach had the lowest H_2_O_2_ among the spinach sample types tested, although these differences were not statistically significant (Table 2).

### 3.4. Relation Between EcO157 Density Change and Levels of Carbohydrates and H_2_O_2_

Glucose and fructose are readily taken up by *E. coli* and serve as primary substrates for growth, while sucrose assimilation is variable among strains [29]. We confirmed that EcO157 ATCC43888 utilized sucrose as the sole carbon source, as it underwent high multiplication rates in an M9 minimal medium containing sucrose as the only substrate. Given the large differences observed in cell concentrations achieved by EcO157 over 5 h in different lysates, as well as significant differences in their carbohydrate concentration (Table 2), simple linear regression analysis was conducted to relate EcO157 density change to their concentration in the lysates. Neither the individual concentrations of glucose (R^2^ = 0.15; *p* = 0.269), fructose (R^2^ = 0.18; *p* = 0.227), or sucrose (R^2^ = 0.06; *p* = 0.499), nor their total combined concentration (R^2^ = 0.15; *p* = 0.266), was significantly related to EcO157 cell density change over 5 h incubation in the various lettuce lysates. This would suggest that simple carbohydrates are not major growth-limiting factors for EcO157 in leaf lysates and likely neither in wounded leaf tissue. The observation that different lettuce types, leaf ages, and baby spinach, all supported EcO157 cell densities of 10^9^ cells/mL lysate within 24 h of incubation at 28 °C (as described in Section 3.1), equivalent to cell densities achieved in rich culture media, suggests that overall nutrient levels in leaf lysates were not a growth-limiting factor for EcO157 in this study. This supports our previously observed EcO157 growth dynamics in romaine lettuce lysates [5]. Similarly, acid stress rather than sugar concentration was the primary determinant of *Listeria monocytogenes* multiplication in fruit juices [30].

**Figure 3:**
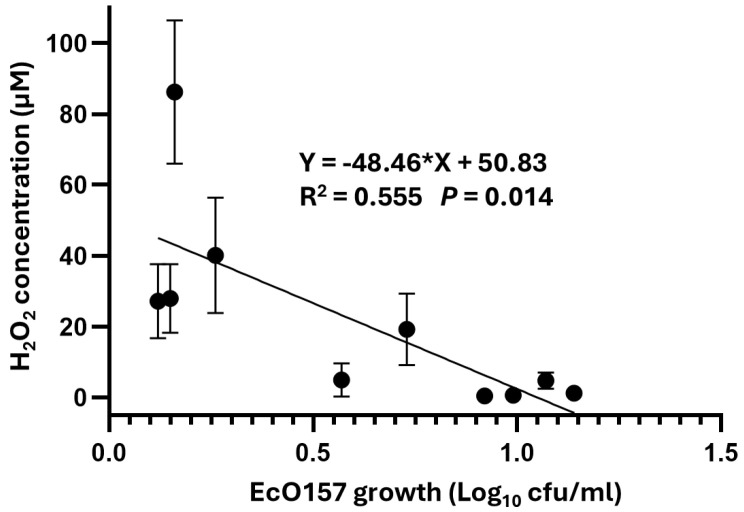
Linear regression of mean H_2_O_2_ concentration over the mean EcO157 density increase over 5 h post-inoculation in the lysate of middle and young leaves of different lettuce types, as per measurements shown in Table 2. *P* refers to the significance of regression slope deviation from a value of zero.

On the other hand, the concentration of H_2_O_2_ in freshly made leaf lysates was significantly inversely related to EcO157 density change, as shown by simple linear regression analysis (Y = −48.46 * X + 50.83; R^2^ = 0.56; *p* = 0.014) (Figure 3). Toivonen et al. [15] reported that physical or chemical removal of H_2_O_2_ at romaine leaf wound sites, which they measured as ranging from 0.62 to 0.98 μM before removal, enhanced the colonization and survival of cut romaine lettuce by EcO157. Thus, the inhibition of EcO157 by the four highest H_2_O_2_ concentrations observed in the middle leaf contents of red leaf, butterhead, green leaf, and romaine lettuce in this study (ranging from 27.13 to 86.18 μM) was expected to inhibit the pathogen as well (Table 2). As reported in Kyle et al. [5], these H_2_O_2_ concentrations and the oxidative stress they imposed were at least partly involved in the longer lag phases of EcO157 observed in middle leaf lysates herein.

We tested for potential multicollinearity of H_2_O_2_ and the other factors in this study that may have affected the outcome of regression analysis. Multiple regression analysis was performed using H_2_O_2_ concentration, leaf age (defined as 6th [young] and 12th [middle] position from within the heart of the lettuce heads), and total sugar concentration as independent variables. EcO157 density change was used as a dependent variable as in the simple linear regression analysis. The results showed variance inflation factor (VIF) values of 1.58, 2.37, and 2.03, and coefficient (R^2^) values of 0.37, 0.58, and 0.51 for the above three independent variables, respectively. These VIF values (between 1.0 and 5.0) and R^2^ values indicate that there is little concern of multicollinearity in our analysis [31].

**Figure 4:**
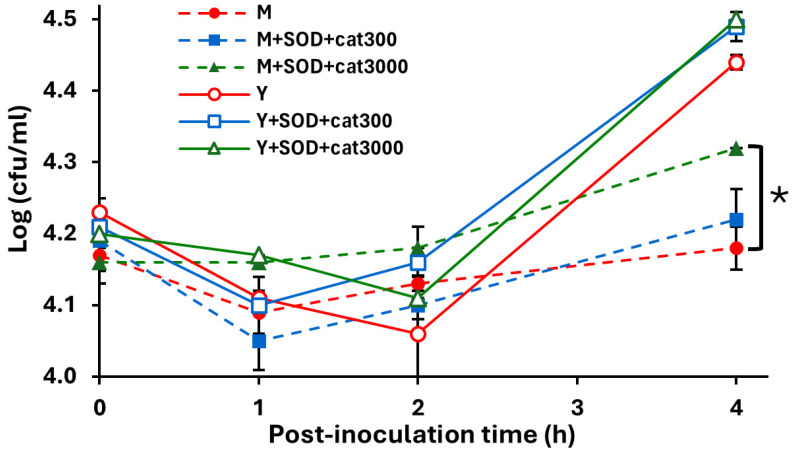
Effect of ROS degradation in romaine lettuce lysates on EcO157 multiplication. EcO157 cell concentrations in the lysates of the middle (dotted lines) and young (solid lines) leaves amended with 150 U/mL SOD and 300 (squares) or 3000 (triangles) U/mL catalase, and without amendment (circles) (control) are shown. Data represent the mean and SEM of two replicate samples from different lettuce heads. * Indicates significant difference in the means of log_10_-transformed population sizes of middle leaves with the amendment of SOD and 3000 U/mL catalase and without amendment (control) (Student’s *t*-test, *p* < 0.05) after 4 h of incubation.

Furthermore, the addition of superoxide dismutase and catalase, two enzymes that degrade ROS, significantly increased the EcO157 cell density achieved over 4 h in the romaine lettuce lysates of middle leaves compared to the non-treated control (Student’s *t*-test, *p* < 0.05) (Figure 4). This enhancement effect occurred only at the higher catalase concentration of 3000 units/mL and only in the middle leaves, which had significantly 5.8-fold greater H_2_O_2_ than the young leaves (Table 2). Khalil and Frank [32] also failed to observe a positive effect of ascorbic acid, an antioxidant that reacts with H_2_O_2_, on EcO157 multiplication when added to lysates of romaine baby lettuce. While other plant constituents may also have negatively impacted EcO157 in our experimental system, our regression analysis clearly reflects that H_2_O_2_ played a significant role in differences among lettuce types and leaf ages.

## 4. Conclusions

A wide range in concentration of simple carbohydrates readily used as substrates by EcO157 was observed among leaves of different ages and lettuce types investigated in this study. Simple sugar concentrations were overall greater in young than middle leaves but did not relate to EcO157 change in density in the leaf cellular contents (lysates) over a 5 h incubation under growth-permissive temperature. H_2_O_2_ concentration was lower in young leaves than in middle leaves of various lettuce types and of spinach. EcO157 density increase under temperature abuse was significantly negatively related to leaf concentration of H_2_O_2_, supporting a role for this bioactive compound in the modulation of EcO157 behavior in leaf contents. As the consumption of fresh-cut leafy greens and of fresh beverages made of homogenized uncooked fruit and vegetables is continually increasing, our results indicate that the sugar content of fresh produce is of lower concern with regard to microbial safety than the maintenance of bioactive compound concentration (for example that of ROS). Efforts should focus on enhancing plant antimicrobial factors to diminish the growth potential of pathogenic bacterial contaminants under temperature abuse in such foods. In light of our study, these efforts may lie also in the prudent use of antioxidants to avoid the browning of produce-derived foods consumed raw in order to preserve ROS. Given current efforts to identify plant traits that render produce less hospitable to foodborne pathogens, H_2_O_2_ content in leafy greens may be of interest in plant breeding programs.

## Figures and Tables

**Table 1:** Strains used in this study.

Strains	Description	Source
*E. coli* O157:H7		
ATCC43888	Non-toxigenic, isolated from human feces	ATCC
RM6067	Isolated from bagged baby spinach in Pennsylvania; 2006 multistate outbreak, USA	Robert Mandrell
RM6069	Isolated from a patient in Pennsylvania; 2006 multistate outbreak, USA	Robert Mandrell
RM6102	Isolated from river water near California spinach field associated with 2006 outbreak, USA	Robert Mandrell
RM6103	Isolated from cow feces near California spinach field associated with 2006 outbreak, USA	Robert Mandrell
RM10002	Isolated from bagged baby spinach in Illinois; 2006 multistate outbreak, USA	Robert Mandrell

**Table 2:** EcO157 growth and concentrations of sugars and H_2_O_2_ in the lysate of middle and young leaves from various lettuce varieties and spinach.

Lettuce/Spinach Type-Leaf Age ^1^	EcO157 Growth ^2^ Log_10_ (cfu/mL)		Total Sugars ^3^ (mg/mL)		H_2_O_2_ Concentration (μM)	
Butter-M	0.26	E ^4^	87.64	D	40.06	B
Butter-Y	1.14	B	455.88	C	1.14	E
Green-M	0.15	E	474.26	C	27.96	BC
Green-Y	0.58	D	1234.63	A	4.89	DE
Red-M	0.15	E	357.78	C	86.18	A
Red-Y	0.72	CD	492.34	C	19.17	BE
Iceberg-M	0.93	BC	362.69	C	0.43	E
Iceberg-Y	0.99	BC	896.47	B	0.61	E
Romaine-M	0.12	E	414.05	C	27.13	BCD
Romaine-Y	1.06	B	849.40	B	4.70	E
Spinach-M	ND		ND		10.66	CE
Spinach-Y	ND		ND		4.88	CE
Spinach-baby	1.67	A	ND		1.07	CE

^1^ Leaf age denoted as M, middle leaf, and Y, young leaf, both from the same plants. ^2^ Increase in cell concentration within five hours of incubation; ND, not determined. ^3^ Total sugars represent total concentration of fructose, glucose, and sucrose. ^4^ Within each column, different letters indicate significant difference in the means (Tukey’s multiple comparisons test, adjusted *p* < 0.05) and alphabetical order indicates values of the mean ranging from highest to lowest.

## Data Availability

The original contributions presented in this study are included in the article. Further inquiries can be directed to the corresponding author.
